# Use of Chinese Herbal Medicine Improves Chemotherapy-Induced Thrombocytopenia among Gynecological Cancer Patients: An Observational Study

**DOI:** 10.1155/2018/4201325

**Published:** 2018-07-12

**Authors:** Yi-Hong Wu, Hsing-Yu Chen, Chyong-Huey Lai, Chein-Shuo Yeh, Jong-Hwei S. Pang, Jian-Tai Qiu, Hung-Hsueh Chou, Lan-Yan Yang, Yu-Bin Pan

**Affiliations:** ^1^Division of Chinese Internal Medicine, Center for Traditional Chinese Medicine, Chang Gung Memorial Hospital, Taoyuan, Taiwan; ^2^School of Traditional Chinese Medicine, College of Medicine, Chang Gung University, Taoyuan, Taiwan; ^3^Gynecologic Cancer Research Center, Chang Gung Memorial Hospital and Chang Gung University College of Medicine, Taoyuan, Taiwan; ^4^Graduate Institute of Clinical Medical Sciences, College of Medicine, Chang Gung University, Taoyuan, Taiwan; ^5^Department of Obstetrics and Gynecology, Chang Gung Memorial Hospital and Chang Gung University College of Medicine, Taoyuan, Taiwan; ^6^Graduate Institute of Traditional Chinese Medicine, Chang Gung University, Taoyuan, Taiwan; ^7^Biostatistics Unit, Clinical Trial Center, Chang Gung Memorial Hospital, Taoyuan, Taiwan

## Abstract

**Background:**

Chemotherapy-induced thrombocytopenia (CIT) is a serious complication among patients with gynecological malignancies, yet management options are limited. This study aimed at reporting the potential of the Chang Gung platelet elevating formula (CGPEF), a prescription with a fixed proportion of Chinese herbs, for improving CIT among gynecologic cancer patients.

**Materials:**

From 1/1/2007 to 31/12/2009, a total of 23 patients with two consecutive CIT episodes (≤ 100×10^3^ /*μ*L) (last cycle: C0; index cycle: C1) received the CGPEF from the nadir of platelet count of C1 and through the subsequent chemotherapy cycles (C2 and beyond). The CGPEF was taken orally four times a day. The evolution of platelet counts of 18 patients after administration of CGPEF was analyzed (2 patients had different chemotherapy regimens after CGPEF, two patients discontinued CGPEF due to the flavor and the amount of CGPEF, and one patient had no further chemotherapy).

**Results:**

Most of the patients had recurrent ovarian cancer (11/18, 61%) with a median of 2.5 previous chemotherapy regimens, and carboplatin-based regimens were the most commonly used for these patients (13/18, 72%). The trend of successive CIT could be reversed after taking CGPEF. Also, the platelet nadir was higher after CGPEF treatment (16.5×10^3^/*μ*L versus 32×10^3^/*μ*L, before and after CGPEF treatment, resp.,* p* = 0.002). Moreover, the chemotherapy interval decreased from 30.5 days to 24 days. No thrombocytosis, clinical bleeding, thromboembolism, or other adverse events were found among these patients.

**Conclusions:**

The CGPEF is worthy of further large-scale, well-designed clinical trials for CIT among gynecological cancer patients.

## 1. Introduction

Thrombocytopenia is one of the most severe complications of chemotherapy, and more than one-fifth of adult patients receiving chemotherapy ever experience thrombocytopenia [1, 2]. In addition to increased bleeding risks, CIT may force physicians to reduce chemotherapy dose, change the chemotherapy regimen, and postpone the chemotherapy schedule, although the treatment is effective [3–5]. These events may prolong hospitalization course, increase medical cost, lower quality of life, and even influence disease outcome [3, 4]. In addition to cisplatin-based chemotherapy, regimens containing carboplatin, paclitaxel, and gemcitabine are regimens highly related to CIT [2]. These drugs are all commonly used for the treatment of gynecological malignancies, such as endometrial, ovarian, and cervical cancer, and therefore CIT becomes an important issue when treating gynecological cancer, especially after several cycles of chemotherapy [6].

Treatment for CIT is still a challenging work of daily clinical practice for oncologists compared to treatment for chemotherapy-induced neutropenia or anemia, which are minimized due to the application of recombinant hematopoietic factors [7]. For instance, among patients with epithelial ovarian cancer, CIT may lower the survival rate due to the 1st-line chemotherapy modification if CIT remains unmanageable [8–10]. In the past decades, several agents have been developed for relieving megakaryocyte depletion, which is the main mechanism of CIT due to direct cytotoxic or immune-mediated effects of chemotherapy drugs. Interleukin-11 (IL-11) is the only agent that is approved commercially in the United States for CIT, but the clinical use is limited due to narrow therapeutic index and significant side effects, such as edema, dyspnea, arrhythmia, syncope, and fatigue [11]. Additionally, some novel thrombopoietic stimulants have also been developed, such as recombinant human thrombopoietin (rhTPO), pegylated recombinant human megakaryocyte growth and development factor (PEG-rHuMGDF), and TPO receptor agonists [5, 12]. The clinical use of rhTPO and PEG-rHuMGDF is limited due to the development of neutralizing antibody [5]. Eltrombopag and romiplostim are two TPO agonists ever used for CIT, but there are only a few small-scale studies about the efficacy in the recent years, and possible thrombocytosis after the medication is of concern [13–15]. Besides, the available weak evidence did not support the use of TPO agonists on CIT among patients with solid malignancies in a recent systemic review and meta-analysis [12]. For this reason, the study about alternative treatments for CIT is still needed.

Traditional Chinese medicine (TCM), one of the most commonly used complementary and alternative medicines in the world, has been used and studied increasingly yearly [16]. In Taiwan, modalities of TCM include Chinese herbal medicine (CHM), acupuncture, and massage, and all these modalities are reimbursed by the national insurance. Nearly a half of cancer patients have used TCM, and more than 90% of cancer patients have been reported to use CHM as an alternative therapy [17–19]. The effectiveness of CHM in relieving side effects due to chemotherapy, such as nausea, vomiting, and fatigue, has been reported in some reports [20–24]. Additionally, some CHM, such as* Astragalus membranaceus*, has been found to be beneficial for neutropenia and anemia after chemotherapy [21, 25, 26]. Nevertheless, to the best of our knowledge, reports about CHM treatments on CIT are still lacking, and the efficacy of CHM remains to be elucidated [24].

This study aims to demonstrate potential efficacy of the Chang Gung platelet elevating formula (CGPEF), a CHM formula with fixed proportion, for a series of patients with gynecological malignancies suffered from CIT. The results of this study provide the basis for further well-designed clinical trials for CGPEF on CIT patients.

## 2. Methods

### 2.1. Study Design

**Figure 1:**
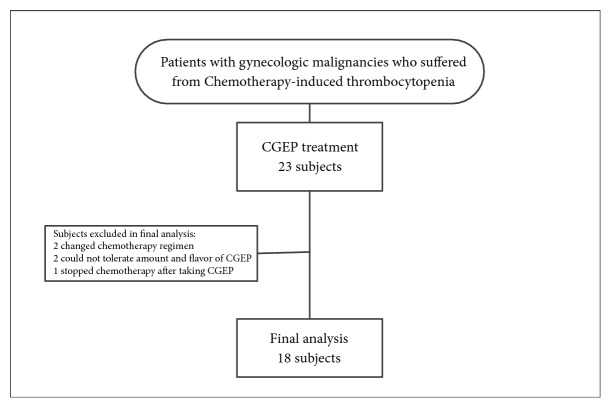
Diagram of enrollment and investigation of subjects in this study.

From 1/1/2007 to 31/12/2009, patients with gynecological malignancies who suffered from two successive episodes of CIT were referred to TCM doctor for the CGPEF treatment. The patients' eligibility is assessed by two gynecologic oncologists (Dr. Chyong-Huey Lai and Jian-Tai Qiu) in the Chang Gung Memorial Hospital (CGMH) once CIT was noted after two successive chemotherapy cycles, while CIT was defined as the platelet count lower than 100×10^3^/*μ*L after chemotherapy. The decision about eligibility was mainly based on gynecologic oncologists' clinical experiences and patients' preference. If CIT had occurred in the previous chemotherapy cycle (C0) and CIT recurred at the subsequent course (C1), the intervention of CGPEF would be suggested and administered from the nadir of platelet count of C1 and through the subsequent chemotherapy cycles (C2 and beyond) if patients agreed (Figure 1).

### 2.2. The Chang Gung Platelet Elevating Formula (CGPEF) for CIT

The CGPEF is a prescription composed of 24 kinds of CHMs from 4 classic formulas: Ren-Shen-Yang-Ying-Tang, Gui-Pi-Tang, Gui-Lu-Er-Xian-Jiao, and Hu-Qian-Wan. These formulas have same ingredients recorded in the TCM textbooks, except tiger bone in Hu-Qian-Wan, because it is strictly prohibited in Taiwan. All CHMs have been commonly used for hundreds of years and were often used by TCM doctors to treat qi, blood, and kidney deficiency, which are thought to be the main cause of CIT on TCM's viewpoint [24, 27]. The composition of the CGPEF is summarized in Table 1. All these herbs are produced by the Chuang Song-Zong Pharmaceutical Factory with good manufacturing practice during the entire observation time and are examined in detail for possible heavy metal, pesticide, or toxin. Every patient was given one pack of CGPEF, weighted 18 gm for adult weighing more than 60 kg, four times a day. The content of CGPEF, safety issues, and possible effectiveness were well explained to these patients in detail after the eligibility was confirmed. Patients were free to choose to take the CGPEF or not or discontinue the CGPEF at any time.

### 2.3. Ethical Consideration

The Institutional Review Board at Chang Gung Memorial Foundation approved this study (No. 99-1241B, 102-0721C).

### 2.4. Outcome Assessment

The changes in platelet counts after taking the CGPEF were the primary outcome of this study, while the lab data of each patient was collected retrospectively. The timing to collect blood samples was between days 7 and 14 of the cycle of chemotherapy in C1 and the first day of next cycle (C2) with 3-5 days' intervals, depending on the severity of thrombocytopenia. Outcome parameters were compared between C1 and C2 (Figure 1). The secondary outcome parameters included duration of platelet counts less than 25×10^3^/*μ*L, 50×10^3^/*μ*L, and 75×10^3^/*μ*L after chemotherapy, platelet recovery time to 50×10^3^/*μ*L and 75×10^3^/*μ*L, and the interval between chemotherapies. Moreover, the need for blood transfusion was recorded as transfusion frequency and amount.

### 2.5. Statistical Analysis

Wilcoxon signed-rank test was used to compare platelet counts, durations, and days for recovery of C1 and C2, presenting the condition of CIT before and after CGPEF treatment. All statistical calculation is done by the SPSS software and the results with* p* value less than 0.05 were thought to be statistically significant.

## 3. Results

**Figure 2:**
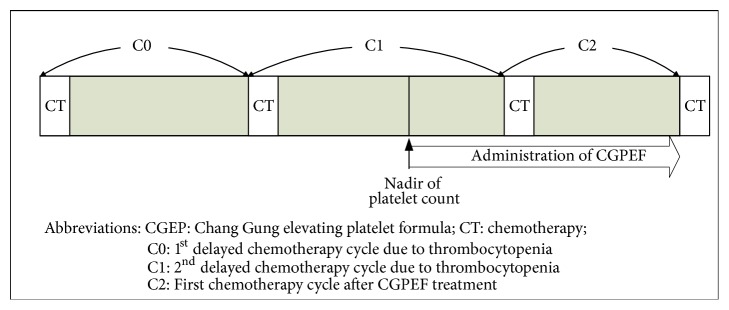
Scheme of managements and follow-up of CGPEF treatment.

A total of 23 patients ever received CGPEF for their CIT, and 18 of them are included in the final analysis. Among the excluded patients, two patients could not tolerate CGPEF due to drug amount and flavor; two patients received different chemotherapy regiments when taking CGPEF and one patient did not receive further chemotherapy after CGPEF treatment (Figure 2). The characteristics of enrolled patients are listed in Table 2. These patients aged 58.06 years on average. More than 60% of patients had ovarian cancer, followed by cervical cancer (27.78%). These patients had no other severe comorbidities, and only one patient had well-controlled hypertension. These patients had regionally or distantly advanced malignancies and had received at least two courses with different chemotherapy regimens for palliative intention (2.72 courses in average). Carboplatin was the most commonly used chemotherapy among these patients, about 72.2% of all patients.

**Figure 3:**
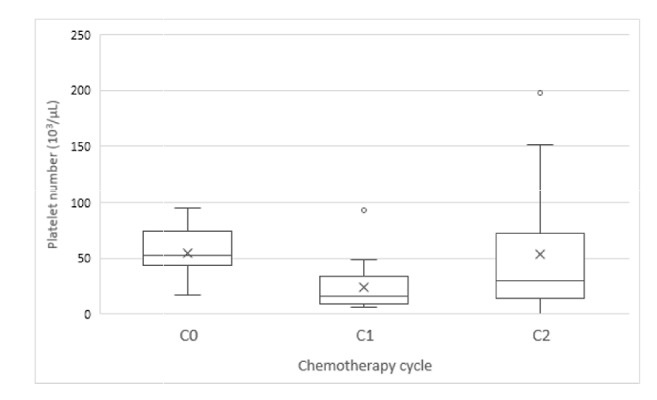
The distribution of nadir platelet count after chemotherapy (C0: the first cycle of nadir platelet count ≤ 100×10^3^/*μ*L; C1: the successive cycle with nadir platelet count ≤ 100×10^3^/*μ*L; C2: the first chemotherapy course completely treated by CGPEF).

**Figure 4:**
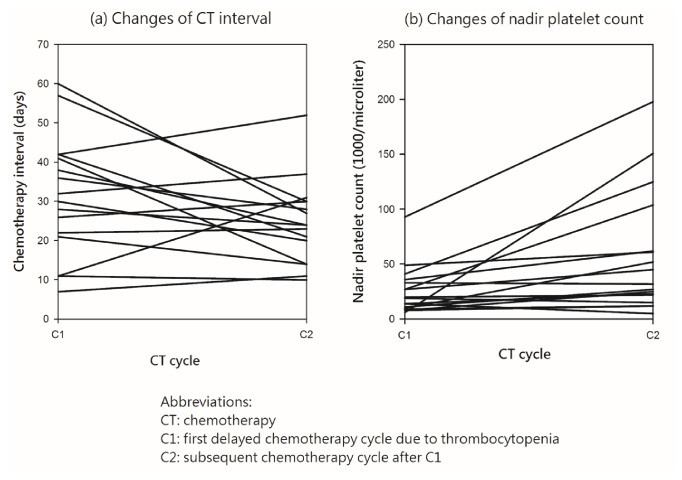
The trend of changes in chemotherapy interval (A) and nadir platelet count (B) before and after CIT treated by CGPEF (C1: the second CIT after chemotherapy; C2: the first chemotherapy course completely treated by CGPEF).

The interval in chemotherapy initiation date extended to 31 days in average (median value: 30.5 days). The nadir of platelet count was about 24.1×10^3^/*μ*L (median count: 16.5×10^3^/*μ*L) (Table 3). After CGPEF treatment, the interval of chemotherapy cycles was about nine days shorter than a previous cycle (30.5 versus 24 days in median value,* p* = 0.109). Additionally, the nadir of platelet count was also higher after treatment (16.5×10^3^/*μ*L versus 32.0×10^3^/*μ*L in median value,* p* = 0.002). However, duration of nadir and recovery time did not differ significantly, although the durations were all shorter after CGPEF treatment (Table 3). Overall, the trends of thrombocytopenia after chemotherapy seemed reversible after CGPEF treatment (Figures 3 and 4), in which median value was 53×10^3^/*μ*L. 62.5% of patients could have nadir higher than 25×10^3^/*μ*L after chemotherapy during CGPEF treatment.

Generally, the CGPEF was well tolerated for the patients, except the intolerance to CGPEF amount and smells. Sometimes patients complained about minor abdominal fullness when taking CGPEF but it recovered soon after adjusting the medication time. As for chemotherapy, hematologic disorders were the most concerned complications after chemotherapy. For leukocyte, one patient had grade IV, five patients had grade III, and seven patients had grade I-II leukopenia, without fever episodes. Additionally, for anemia, one had grade III anemia, and the other 17 patients ever experienced grade I-II anemia, according to the Common Terminology Criteria for Adverse Events v3.0.

## 4. Discussion

CGPEF may be a potentially effective alternative treatment for CIT with the significantly higher nadir of platelet count after/before CGPEF among patients with advanced gynecological malignancies. Also, CGPEF seems beneficial to improve delays in subsequent chemotherapy with marginally statistical significance. The benefit of CHM has been proposed for treating chemotherapy-related side effects; however, the evidence is still lacking, especially for CIT patients with gynecological malignancies [22]. Thrombocytopenia is one of the most important factors to postpone patient's subsequent chemotherapy since carboplatin is commonly used among these patients, as our case series [28]. Delayed chemotherapy, especially with a reduced dose, could decrease overall survival rate [8–10]. Additionally, thrombocytopenia may increase risks of gastrointestinal bleeding and even hemorrhagic stroke [1]. It is difficult in reversing the trend of CIT if chemotherapy is ongoing without any intervention in clinical practice. CGPEF appeared to reverse the trend of CIT even if chemotherapy was kept without a reduced dose and prolonged interval (Figure 3). The interval between chemotherapies could be shortened to near 21 days, which is the modest interval of chemotherapy, and the median platelet nadir became higher than 30×10^3^/*μ*L, (C2) which is associated with much lower risks for spontaneous bleeding compared to 16.5×10^3^/*μ*L in previous chemotherapy cycle (C1).

Also, to reverse the trend of CIT, the benefit of CGPEF seems comparable to current medical treatment. IL-11, the only drug for CIT which is approved by the Food and Drug Administration in the United States, can elevate nadir of platelet count from 40×10^3^/*μ*L to 60×10^3^/*μ*L, while rhTPOs have been reported to elevate nadir (from 20×10^3^/*μ*L to 44×10^3^/*μ*L) and shorten the duration of thrombocytopenia [11, 29, 30]. Moreover, through stimulating megakaryocyte, platelet transfusion can be reduced by using IL-11 without a reduction in chemotherapy dose [11, 31]. In our case series, the tendency for the duration of and transfusion rate of CIT to decrease can also be found, although not statistically significant (Table 3). The duration of platelet counts less than 50×10^3^/*μ*L and 75×10^3^/*μ*L can be shortened 2-3 days, and it takes 1-2 days shorter to recover to a safe level of thrombocytopenia. This trend may be more prominent if CGPEF starts on the first day of the C2 cycle but not in the late period of the C1 cycle, which may improve recovery of CIT in C1 cycle. Nevertheless, the larger study population is still needed to stress the difference in duration and recovery rate of CIT.

Furthermore, CGPEF treatment was quite tolerable among these patients, and only two patients stopped taking CGPEF due to its flavor and amount (72gm/day). In clinical settings, CGPEF is unfavorable for CIT patients with adhesion ileus or massive ascites. For IL-11, although effective, the drug-related adverse events rate is as high as 30-50%, including edema, fever, hepatitis, coagulopathy, fatigue, and arthralgia [32, 33]. On the other hand, TPO receptor agonists with potential effects on CIT, such as eltrombopag and romiplostim, are reported to increase the risk of cerebral and portal vein thrombosis, abnormal liver function and, most seriously, myelofibrosis [34–36]. Thrombocytosis associated with the use of TPO receptor agonist became another potential drawback, since thrombocytosis may be related to poor prognosis of malignancies [37, 38]. In the light of significantly higher serum TPO level among CIT patients [39, 40], medical treatment other than recombinant TPO and TPO receptor agonist may be needed to recover thrombocytopenia without causing possible thrombosis, myelofibrosis, and even thrombocytosis [7].

CGPEF with potential effects on CIT may be related to megakaryocyte stimulation and stem cell proliferation/differentiation [41]. The precise mechanism may be different from TPO agonist. In TCM theory, a patient with fatigue, nausea, and bone marrow suppression after chemotherapy is often diagnosed as qi and blood deficiency [27]. CGPEF was composed of 24 CHM to supplement qi and blood.* Panax ginseng*, one of the major ingredients of CGPEF, was usually used to supplement qi and its extract, panaxadiol saponins, was able to stimulate megakaryocytic maturation [42]. Moreover,* Astragalus membranaceus* and* Angelica sinensis* radix were reported to improve bone marrow stem cell proliferation [43]. In addition to improvements on thrombocytopenia, the anticancer and immunomodulation effects of* Angelica sinensis [44], Panax ginseng *[45–47], and* Astragalus membranaceus *[48, 49] in bench studies may imply the valuable role as adjuvant therapy in patients with gynecological malignancies and CIT.

In this observational study, we demonstrated the potential efficacy of CGPEF in treating CIT. However, there are still limitations. First, the post hoc power analysis showed that the *β*-error probability was about 0.23 by using Lehmann equation [50], and therefore the conclusion may be more solid if the case number could be higher. However, the important information about medication dose and duration, follow-up duration, examination scheme, and even endpoints provided in this study is crucial for further large-scale, randomized clinical trials, which are still lacking so far [12]. Second, since this is a pilot retrospective study, the differences in hemogram check-up timing may inevitably cause some errors in recording the timing and the platelet nadir count. However, because gynecologists often check hemogram earlier and more frequently once thrombocytopenia occurs, as two consecutive episodes of platelet count lower than 100×10^3^/*μ*L in C0 and C1 in this study, the error should be minimal. Third, since this is an observation study, we cannot completely exclude the possibility that CIT recovered by nature course. However, since only patients with two successive CIT episodes were included in this study and CIT is highly associated with courses of chemotherapy [1], the spontaneous recovery of CIT seemed less possible.

## 5. Conclusion

In this study, we demonstrated that CGPEF is a potentially effective treatment for CIT. Although this study is relatively small without untreated control, we demonstrated that the median platelet nadir count after CGPEF treatment (C2) was significantly higher than before (C1). Further well-designed, double-blind, placebo-controlled clinical trial with a larger number of subjects can be done based on these preliminary results.

## Figures and Tables

**Table 1:** Composition of Chang Gung platelet elevating formula (CGPEF).

Chinese herbal products	Weight (gm)
*Cervus nippon *Temminck	1.8
*Trionyx sinensis *(Wiegmann)	1.6
*Phellodendron chinense *Schneid.	0.7
*Angelica sinensis *(Oliv.) Diels.	0.7
*Panax ginseng *C. A. Meyer	0.7
*Paeonia lactiflora *Pall.	0.7
*Achyranthes bidentata *Blume	0.6
*Zingiber officinale *Rosc.	0.6
*Rehmannia glutinosa *(Gaertn.) Libosch.	0.5
*Astragalus membranaceus *(Fisch.) Bunge	0.5
*Atractylodes macrocephala *Koidz.	0.5
*Poria cocos *(Schw.) Wolf	0.5
*Polygala tenuifolia *Willd.	0.4
*Glycyrrhiza uralensis *Fisch.	0.4
*Citrus reticulata *Blanco	0.4
*Anemarrhena asphodeloides *Bunge	0.4
*Zizyphus jujuba *Mill.	0.3
*Euphoria longan* (Lour.) Steud.	0.3
*Zizyphus jujuba *Mill.	0.3
*Cinnamomum cassia *Blume	0.2
*Lycium barbarum *L.	0.2
*Schisandra chinensis *(Turcz.) Baill.	0.2
*Cynomorium songaricum *Rupr.	0.2
*Aucklandia lappa *Decne.	0.1

*∗*Each CGPEF contains 24 Chinese herbs (12.7 gm) and 5.3 gm starch, totally 18 gm; daily dose: 72 gm.

**Table 2:** Characteristics of the patients treated with Chinese herbal medicine formula: Chang-Gung platelet elevating formula (CGPEF).

	Count (%)	Median (Q1; Q3)
Age		59.63 (49.11;64.75)
Cancer type		
Cervical cancer	5 (27.8)	
Ovarian cancer	11 (61.1)	
Endometrial cancer	2 (11.1)	

Comorbidities		
Hypertension	1 (5.6)	
Diabetes mellitus	0 (0)	
Ischemic heart disease	0 (0)	
Cerebral vascular disease	0 (0)	
Coagulopathy/bleeding disorder	0 (0)	
Other malignancies	0 (0)	

Preceding chemotherapy regimens		2.5 (1.0;4.0)
Chemotherapy regimens for CGPEF treatment		
Cisplatin-based	2 (11.1)	
Carboplatin-based	13 (72.2)	
Taxane alone	1 (5.6)	
Others	2 (11.1)	

**Table 3:** Comparisons of presentations of CIT before and after using CGPEF (*n* = 18).

	Before CGPEF		After CGPEF		*p* value
	Median	Q1; Q3	Median	Q1; Q3	
Chemotherapy interval (days)	30.5	21.75;41.25	24	15.5;.30.0	0.109
Blood transfusion					
Times (per cycle)	0	0;0	0	0;0	0.564
Amount (units per cycle)	0	0;0	0	0;0	0.999
Platelet counts					
Nadir (10^3^/*μ*L)	16.5	8.75;33.75	32	18.5;83.0	0.002*∗*
< 25×10^3^/*μ*L duration (days)	4	0;7.5	0	0;8	0.999
< 50×10^3^/*μ*L duration (days)	8.5	4.0;13.0	8	0;14.5	0.211
< 75×10^3^/*μ*L duration (days)	14	8.5;28.0	10.5	0.5;19.75	0.279
Recover to 50×10^3^/*μ*L (days)	9	5.5;11.5	10	0;13.0	0.232
Recover to 75×10^3^/*μ*L (days)	10.5	6.75;13.5	10.5	0.5;14.5	0.629
Recover to 100×10^3^/*μ*L (days)	11.5	9.88;19.5	16.25	10.25;24.17	0.050*∗*

*∗* indicates *p* value < 0.05.

CIT: chemotherapy-induced thrombocytopenia.

## Data Availability

The data used to support the findings of this study are available from the corresponding author upon request.
